# The Gly103Arg variant in hereditary transthyretin amyloidosis

**DOI:** 10.3389/fneur.2024.1471131

**Published:** 2024-09-09

**Authors:** Yihan Xiong, Gongcheng Qu, Xiaoyu Lu, Xin Chang, Miaoping Zhang, Jiantang Liang, Kexing Lin, Xiaoman Zhao, Xuejun Fu, Ying Huang, Qianhui Xu

**Affiliations:** ^1^The Second Clinical Medical College, Jinan University, Shenzhen, China; ^2^The First Affiliated Hospital, Southern University of Science and Technology, Shenzhen, China; ^3^Department of Respiratory and Critical Medicine, Shenzhen People’s Hospital (The Second Clinical Medical College, Jinan University, The First Affiliated Hospital, Southern University of Science and Technology), Shenzhen, China; ^4^Department of Neurology, Shenzhen People’s Hospital (The Second Clinical Medical College, Jinan University, The First Affiliated Hospital, Southern University of Science and Technology), Shenzhen, China

**Keywords:** hereditary transthyretin amyloidosis, p.G103R variant, peripheral neuropathy, vitreous opacity, Chinese population

## Abstract

**Background:**

Hereditary transthyretin amyloidosis (ATTRv) is an autosomal dominant inherited systematic disease primarily affecting the peripheral and autonomic nervous system, heart, eyes and kidney. Over 140 variants have been identified worldwide, with the Gly103Arg variant reported exclusively in China. This variant is characterized by early onset eye manifestations, making accurate and timely diagnosis difficult. Therefore, we conducted a case study and literature review to investigate the clinical characteristics of the Gly103Arg variant in hereditary transthyretin amyloidosis.

**Methods:**

We identified three patients and an asymptomatic carrier in a four-generation family by sequencing the TTR gene. The proband underwent a lumbar puncture, electromyography, abdominal fat biopsy, among other tests. Case reports of Gly103Arg variant were retrieved through a literature search for an analysis of clinical characteristics.

**Results:**

The study included clinical data of 44 patients. Our literature review collected data on 41 patients and the present report supplied 3 patients with the Gly103Arg variant. The mean age at onset was 39.1 ± 4.27 years (range 30–47 years) with a female ratio of 52.3%. All cases were reported in China, predominantly in southern regions, especially Yunan and Guizhou Provinces. The initial manifestation was blurred vision, except for one case presenting with numbness in the upper extremities. All of them had vitreous opacity; 17 cases had peripheral neuropathy,6 cases had autonomic neuropathy, and 3 cases had cardiopathy. No disease-related deaths have been reported to date.

**Conclusion:**

The Gly103Arg variant is unique to the Chinese population, frequently occurring in southern China. The main clinical manifestations are blurred vision, vitreous opacity, and neuropathy, with cardiopathy being rare. ATTRv should be considered if a patient diagnosed with CIDP does not respond to related therapy. Abdominal fat biopsy is a convenient and accurate diagnostic method.

## 1 Introduction

Hereditary transthyretin amyloidosis (ATTRv) is an autosomal dominant inherited systemic disease affecting the eyes, peripheral and autonomic nervous systems, heart and kidneys. Over 140 variants have been reported worldwide, including Val30Met, Val122Ile, Thr60Ala, and Ala97Ser. The Val30Met variant is the most frequent and is primarily reported in Portugal, Sweden and Japan (1). TTR is a transport protein synthesized by the liver, choroid plexus of the brain, and retinal pigment epithelium. Variants cause the tetrameric structure become unstable, leading to monomer unfolding and amyloid protein deposition in the tissues of the eyes, nerves, heart, and kidneys (2). The Gly103Arg variant has only been reported in Chinese individuals and is characterized by early onset vitreous opacity and a family history of the disease. Extraocular symptoms are usually mild and occur several years after the ocular manifestations. Here, we report three patients and an asymptomatic carrier in a four-generation family. The proband was a 48-year-old male diagnosed with ATTRv due to the Gly103Arg variant, who had severe peripheral neuropathy. We also reviewed the literature on this variant to analyze its clinical characteristics.

## 2 Materials and methods

### 2.1 Sequencing and genetic analysis

Blood samples were collected from the proband and families, and genomic DNAs were extracted. Briefly, Genomic DNA was firstly sheering into fragment and purification, then captured by the XGen Exome Research Panel (IDT, United States), and finally the libraries were sequenced on the NovaSeq 6,000 Sequencing platform. After removing low-quality reads and adaptors, the paired-end clean reads were mapped with the human reference genome (GRCh38/hg38) by Burrows-Wheeler Aligner (BWA). Variants calling was performed by Genome Analysis Toolkit (GATK). Variants located in exon or classical alternative splicing region with low frequency (<0.01) in public database were obtained for further annotation with ANNOVAR. Pathogenicity of variants were classified according to ACMG (American College of Medical Genetics and Genomics) guidelines. The variants were further validated by Sanger sequencing in all available family members. The nomenclature used standard numbering beginning at the Met initiation codon, which had a 20-amino-acid difference with historic nomenclature reported in older literature (e.g., p.G103R would be referred to as p.G83R).

### 2.2 Case presentation

**Figure 1:**
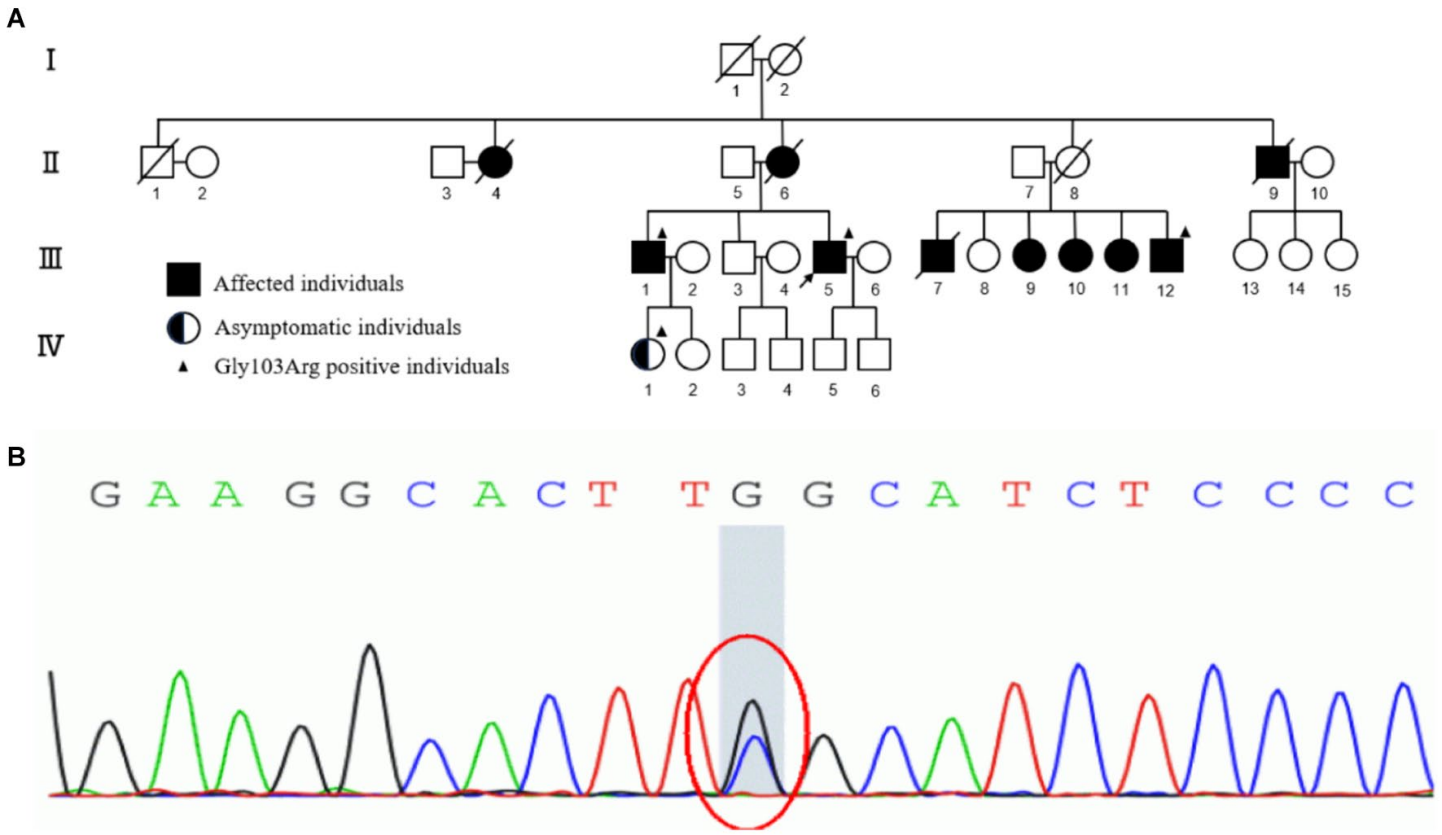
**(A)** The pedigree of the four-generation family. I, II, III, IV represent first, second, third and fourth generation, respectively. Normal individuals are shown as clear circles (females) and squares (males), affected individuals are shown as filled symbols, asymptomatic individuals with the TTR p.G103R are shown as half-filled symbols. The individuals passed away are shown as slash. The arrow indicates the proband. The individuals who underwent genetic analyses are marked by solid triangle. **(B)** Sanger sequencing of exons of TTR data shows that a G to C transversion (red circle) resulted in the substitution of glycine-103 by arginine (Gly103Arg) in affected individuals of the family.

The proband (III-5, Figure 1A) was a 48-year-old male, admitted to the hospital due to gradual limb weakness over 3 years, which had worsened in the past month. Three years ago, he developed numbness and weakness in both hands and below the knees for no apparent reason, progressively leading to difficulties in typing and walking. One month before admission, he was admitted to a local hospital due to aggravated limb weakness and dyspnea. He had a history of osteomyelitis resection over 20 years ago and vitrectomy 6 years ago. A lumbar puncture was performed and CSF examination revealed elevated protein levels (1,008 mg/L) and positive globulin. The serum anti-GT1a antibody was positive, but serum and CSF antibodies against the node of Ranvier CSF ganglioside antibodies were negative. Blood tests were negative for immunoglobulin, antinuclear antibody, rheumatoid factor, tumor markers, syphilis antibody, HIV antibody, spinal muscular atrophy genomic test. Brain and whole spinal MRI showed no abnormalities. An ultrasonic cardiogram indicated decreased left ventricular diastolic function. The proband was diagnosed with chronic inflammatory demyelinating polyneuropathy (CIDP) and treated with pulse therapy of methylprednisolone (1 g/day for 3 days, with a decreasing dosage every 3 days), followed by oral methylprednisolone (56 mg/day). However, the symptoms did not improve significantly.

Upon admission, physical examination revealed reduced muscle volume in the extremities, significant atrophy in the thenar, hypothenar, and interossei muscles of both hands, as well as in the biceps femoris and quadriceps femoris muscles of both lower extremities. Motor testing showed 4/5 strength in the upper extremities and 5−/5 in the lower extremities. Superficial sensation was reduced above 10 cm from the wrists and ankles. Deep tendon reflexes were not elicited, with no other notable neurological abnormalities in the tone of muscle groups, extraordinary sensation and combined sensation. The proband did not have any autonomic dysfunction, such as diarrhea, constipation, dyspareunia, dry mouth, dry eyes, erectile dysfunction. His body mass index was 20.5. Laboratory examinations revealed an increased erythrocyte sedimentation rate (71 mm/L), elevated CK-MB level (29.9 U/L), decreased serum albumin (33.9 g/L), and increased free thyroxine (23.44 pmol/L). Other tests, including complete blood cell count, urinalysis, coagulation, NT-proBNP, glomerular filtration rate, serum creatinine, blood urea nitrogen, folic acid, vitamin B12, HBV antibody, HCV antibody, HIV antibody, syphilis antibody, immunoglobulin G, serum protein electrophoresis, immunofixation electrophoresis, urinary protein quantitation, and urine immunoglobulin electrophoresis, were negative.

**Figure 2:**
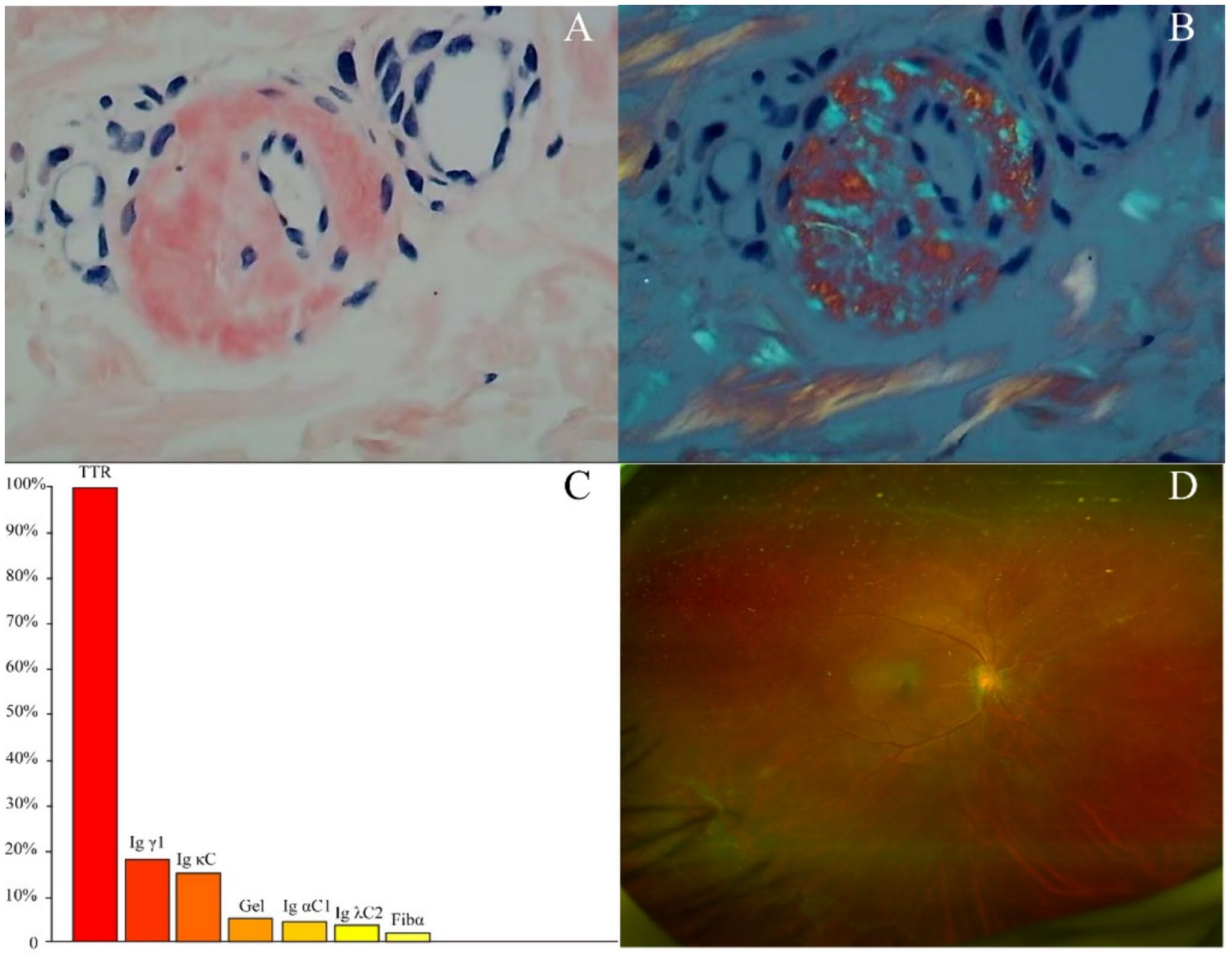
**(A)** Congo-red staining of the abdominal tissues revealed pale red unstructured materials in the superficial dermis and arteriolar wall. **(B)** The Congo-red positive materials were also observed with “apple-green” under polarized light. **(C)** Amyloidosis mass spectrometry analysis of abdominal tissues showed that TTR had the highest relative abundance. **(D)** Fundus examination showed vitreous opacities, cotton wool-like deposits on the retina around the optic disk.

The proband’s diopter in the right eye was spherical power of-1.25 diopters. The patient’s diopter in the left eye was spherical power of-4.75 diopters and-0.50 diopters of cylinder at 35 degrees. Anterior segment slit-lamp examination and fundus examination after pupillary dilatation revealed vitreous opacities, cotton wool-like deposits on the retina around the optic disk, and old laser spots on the temporal side and below the peripheral retina of the left eye (Figure 2D). Optical coherence tomography showed a burr-like hyperreflective signal on the retinal surface of both eyes. Intraocular pressure, macular structure, and retinal nerve fibre layer were generally normal in both eyes. Nerve conduction studies showed severe chronic multiple peripheral neuropathy involving movement and sensation, with axonal involvement being the most serious symptom. There was no obvious abnormalities in brain contrast-enhanced MRI, contrast-enhanced magnetic resonance angiography, cervical contrast-enhanced MRI, 24-h ambulatory electrocardiogram, head-up tilt test, abdominal ultrasound, and cognition assessment.

The proband had gradual limb weakness for 3 years, with CSF examination showing increased protein levels and normal nucleated cell counts. Additionally, his serum anti-GT1a IgG was positive. However, he did not exhibit common manifestations of GBS with anti-GT1a IgG, such as ophthalmoplegia, throat and neck muscle weakness, and sensation abnormalities. Given his lack of improvement after methylprednisolone pulse therapy, history of vitrectomy, and family history of vitreous opacities and lower extremity weakness, he was likely diagnosed with ATTRv-PN.

Two tissue samples from the proband’s abdominal fat were obtained for biopsy and amyloidosis mass spectrography. Congo-red staining of the abdominal tissues revealed pale red unstructured materials in the superficial dermis and arteriolar wall with “apple-green” under polarized light, indicating amyloid deposits (Figures 2A,B). Amyloidosis mass spectrometry analysis of abdominal tissues showed that TTR had the highest relative abundance (Figure 2C). Sanger sequencing of the TTR gene revealed a heterozygous variant c.307G > C (p.G103R) in the proband (III-5) (Figure 1). The proband was subsequently diagnosed with ATTRv and treated with tafamidis (20 mg/day), which stabilizes TTR–tetramer protein structure and reduces TTR-variant protein aggregation. During a 3-year follow-up, the proband was treated with tafamidis (20 mg/day) regularly without symptom aggravation.

The proband’s mother (II-6), maternal uncle (II-9), and eldest brother (III-1) had a history of vitreous opacity and lower extremity weakness, while his maternal aunt (II-4) and five cousins (III-7,9–12) had a history of vision loss. His niece (IV-1) did not have any symptoms. His oldest brother (III-1), his cousin (III-12), and his niece (IV-1) also underwent sanger sequencing and were detected the same variant c.307G > C (p.G103R) as the proband. Regrettably, they did not get further examination due to transportation difficulties (Figure 1A).

### 2.3 Literature review

A literature search was conducted using the PubMed database. The following combinations of search terms were used: “Gly83Arg”, “Gly103Arg”, “p.G83R”, “p.G103R” and “c.307G>C”. The search was limited to articles in English. Available data in the form of abstracts or full-text articles, along with related citations and references, were reviewed. Clinical data of 41 patients with the Gly103Arg variant from 18 unrelated families were collected from 9 literature sources (2–10).

## 3 Results

This variant has been documented in several studies. Therefore, to gain a comprehensive understanding of the clinical manifestations and auxiliary findings associated with this condition, we conducted a thorough review of the relevant literature and extracted data from the medical records of 41 patients. Additionally, we included data from the patients reported in this paper. The results of the literature review and the present cases are shown in Table 1. Of the 44 patients included in the study, 21 were male and 23 were female, with a mean age of onset of 39.1 ± 4.27 years (range 30–47 years). All patients were from China, primarily from the southern region, with Guizhou being the most common location. The initial clinical manifestations were primarily blurred vision, with only one case presenting with numbness of the upper limbs (8). All patients had a family history of vitreous opacity and underwent ophthalmological examination.

**Table 1:** Characteristics of patients with symptomatic Gly103Arg-related ATTRv in this study and reviews.

Characteristic	Counts (n = 44)	Ratio (%)
Southern China	38	86.4
Female	23	52.3
AO (years)	39.1 ± 4.27 (range 30–47 years)	/
Family history	44	100
VO onset	43	97.7
PN onset	1	2.3
onset to SMPN (years)	7.2 ± 5.50 (range 3–15 years)	/
VO	44	100
PN	17	53.1
AN	6	13.7
Cardiac	3	10.7
Death	0	0

AO, age of onset; VO, vitreous opacity; PN, peripheral neuropathy; SPMN, sensory-motor polyneuropathy; AN, autonomic neuropathy.

A total of 32 patients underwent a neurological examination, with 17 (53.1%) exhibiting signs of peripheral nerve damage. Ten patients underwent electromyography, with eight (80%) demonstrating sensory-motor polyneuropathy. Of these, three were female and five were male. The mean interval between the onset of the disease and the diagnosis of sensory-motor polyneuropathy (SMPN) was 7.2 ± 5.50 years (range 3–15 years). Six patients presented with autonomic nervous system symptoms, two of whom had a positive upright tilt test result. A total of 28 patients underwent cardiac examinations, with three (10.7%) exhibiting cardiac hypertrophy, one of whom also exhibited atrioventricular block. No fatal cases have been reported (Table 1).

The most common clinical manifestation of this variant was ocular symptoms, with all patients presenting with a history of blurred vision and a complete examination suggestive of vitreous opacity and degeneration. Vitreous opacity could be unilateral or bilateral, and most patients had a history of recurrence after vitrectomy. Other ocular structures, including the lens, iris, and retina, have also been reported to be involved (5). Funduscopic examination, ocular ultrasound, and scanning laser fundus examination (SLO) can be employed to identify vitreous opacity (9).

The principal symptoms of the peripheral nervous system are carpal tunnel syndrome and sensorimotor polyneuropathy. Electromyography may reveal decreased neurosensory-motor conduction velocity, decreased action potential amplitude in innervated muscles, impaired neurosensory conduction, and axonal damage. A comprehensive search of the literature and the EMG data of this case revealed that these patients exhibited a notable decrease in action potential amplitude and a slowing of nerve conduction velocity. The sensor damage was more sever than the motor damage. Furthermore, the amplitude of action potentials exhibited a more pronounced decline, suggesting that axonal damage was a predominant feature. As the variant was usually reported by ophthalmology department, electromyography was underwent in only a few patients and the peripheral nervous abnormalities might be underestimated (Table 2). Ultrasound imaging of the nerve revealed a thickening, predominantly in the proximal and carpal tunnel regions.

**Table 2:** Neuroelectrophysiological features of patients with symptomatic Gly103Arg-related ATTRv in this study and reviews.

Nerves	Parameters	1	2	3	4	5	6	7
Median motor nerves	DML,ms	**6.6**	3.9	**4.5**	**6.1**	3.1	3.5	**8.36**
	CMAP,mV	**2.9**	10.4	7.9	**1.9**	**4.2**	7.1	**2.5**
	MCV,m/s	51.2	59.5	54.3	**47.5**	52	**49.7**	**46.5**
Ulnar motor nerves	DML,ms	2.8	2.7	2.7	**3.7**	2.7	2.7	**8.30**
	CMAP,mV	6.5	9.4	7.9	**4.0**	10.6	9.7	**0.55**
	MCV,m/s	52.4	57.8	50.5	**41.7**	57.8	52.0	**48.7**
Peroneal nerves	DML,ms	4.5	4.1	4.5	**6.6**	**NR**	3.7	**13.0**
	CMAP,mV	3.9	4.5	5.6	**0.2**	**NR**	**2.0**	**1.35**
	MCV,m/s	45.4	49.3	45.5	**34.9**	**NR**	**38.9**	**35.8**
Tibial nerves	DML,ms	3.7	4.0	3.7	**6.9**	**NR**	4.9	**13.1**
	CMAP,mV	4.7	7.8	10.2	**0.2**	**NR**	**3.2**	**5.9**
	MCV,m/s	42.4	48.6	47.0	**38.2**	**NR**	40.1	**40.5**
Median sensory nerves	SNAP,μV	**NR**	**5.0**	**NR**	**NR**	**3.0**	5.8	**4.7**
	SCV,m/s	**NR**	**34.3**	**NR**	**NR**	**46.8**	**49.4**	**48.4**
Ulnar sensory nerves	SNAP,μV	**NR**	8.9	**NR**	**NR**	10.6	5.2	**NR**
	SCV,m/s	**NR**	**48.2**	**NR**	**NR**	57.8	**47.6**	**NR**
Superficial peroneal nerves	SNAP,μV	**1.9**	8.1	6.3	**NR**	ND	13.0	**NR**
	SCV,m/s	**38.8**	52.0	47.3	**NR**	ND	49.2	**NR**
Sural nerves	SNAP,μV	**NR**	6.3	5.0	**NR**	ND	6.0	**2.0**
	SCV,m/s	**NR**	51.5	48.9	**NR**	ND	41.1	**35.9**

1–6 were referenced from (2); 7 was proband in this study. CMAP, compound muscle action potential; DML, distal motor latency; MCV, motor conduction velocity; ND, not done; NR, not record; SCV, sensory conduction velocity; SNAP, sensory nerve action potential. The abnormal values were printed in bold.

Amyloid deposits, as evidenced by Congo red staining, has been observed in various tissues, including the vitreous, conjunctival, neurological, and muscular tissues (10). Autonomic symptoms include diarrhea, constipation, alternating diarrhea and constipation, dyspareunia, dry mouth, dry eyes, erectile dysfunction, change in skin color, and postural dizziness, with a positive upright tilt test. Cardiac lesions are characterized by the development of cardiac hypertrophy and atrioventricular block.

## 4 Discussion

We identified three patients and an asymptomatic carrier in a four-generation family by sequencing the TTR gene. The proband underwent a lumbar puncture, electromyography, abdominal fat biopsy, and genomic sequencing. Eventually, the proband was diagnosed with ATTRv due to the Gly103Arg variant. It was the most common genotype in the Chinese population, which most likely presented visual and neurological abnormalities without reported death. Other genotypes such as Val30Met, Val30Al, Leu55Arg, Ala36Pro, Lys35Thr, Gly47Arg, Asp18Gly, Gly47Glu, Ala117Ser were also reported in the Chinese population (11).

Hereditary transthyretin amyloidosis is an autosomal dominant inherited systematic disease caused by TTR gene variants. Over 140 variants have been detected to date, and the incidence of different variants may have correlate with ethnicity and geography. For example, the Val30Met variant is frequently reported in Portugal, Sweden and Japan, while Val122Ile is seen in African Americans, West Africans, and Spaniards. The Thr60Ala variant usually occurs in Northern Ireland, and the Ala97Ser variant occurs in China and Taiwan (1). The Gly103Arg variant reported in this case has only been documented in China, appearing to be specific to the Chinese population, with a high prevalence in the southern region, especially in Yunnan and Guizhou. Additionally, there were no fatal cases been reported. We speculated that was because of the less cardiac dysfuction compared to other variants.

ATTRv variant exhibit various different phenotypes, including neurologic, cardiac, and mixed, forms. For instance, early-onset Val30Met often manifests as peripheral neuropathy, Val122Ile as cardiac issues, and late-onset Val30Met with both peripheral neurologic and cardiac manifestations (12). The variant described in this article typically begins with ocular symptoms, such as blurred vision and vitreous opacity, with other systemic symptoms either absent or not obvious. This can lead to misdiagnoses as simple vitreous degeneration, prompting initial treatment with vitreous surgery. However, patients often experience recurring blurred vision, vitreous degeneration, and other complications, with peripheral, autonomic, and cardiac manifestations emerging as the disease progresses. This delay in diagnosis can lead to inadequate treatment and a decline in the patient’s quality of life. Also, there were many other genotypes presenting visual abnormalities, such as Glu54Gly, Val30Met, Glu89Lys, Asp18Glu, Ala36Pro, Tyr114Cys (13).

From the literature, we found that early diagnosis can be aided by certain clues. The age of onset for this variant is relatively early, between 30 and 50 years. All reported cases have a family history, and most are from southern China, especially Yunnan and Guizhou. As such, clinicians should inquire about the patient’s place of origin and family history of blurred vision, limb numbness or weakness, carpal tunnel syndrome, or heart disease. While liver transplantation is effective for ATTRv-PN, it does not address ocular and cardiac lesions. Furthermore, tafamidis may not achieve therapeutic effect due to its low concentration in the blood-ocular barrier. Therefore, surgical treatment remains the primary option for ocular lesions (3). It is recommended that surgical histopathological examination be performed immediately after the first vitrectomy in patients with high suspicion of ATTRv to facilitate early diagnosis and treatment.

Despite the predominance of the ocular symptoms in this variant, peripheral neurologic symptoms are also common, and patients may present with limb numbness and weakness. As the number of TTR variants continues to grow, clinicians are becoming increasingly aware of ATTRv; however, the misdiagnosis rate remains high, particularly as CIDP (14). As detailed in this study, the patient was referred for progressive limb weakness. A lumbar puncture suggested protein cell separation, and serum anti-GT1a antibody testing returned a positive result. Electromyography indicated that the peripheral nerves of the limbs exhibited severe chronic multiple peripheral neuropathic lesions, which aligned with the diagnostic criteria for CIDP. However, methylprednisolone pulse therapy demonstrated limited efficacy, prompting consideration of the differentiation between CIDP and ATTRv.

The phenomenon of CSF protein cell separation has been observed in some ATTRv patients. Analysis of the relevant literature suggests that this may be due to an increase in protein content from the accumulation of transthyretin protein, which is produced by the choroid plexus and resistant to degradation due to amyloidosis. Additionally, myelin thickening may be identified in neuropathological examinations (2). It is proposed that a reparative process analogous to CIDP may occur, whereby myelin proteins are transported into the CSF, leading to elevated protein concentration. The obstruction of CSF circulation due to various factors can also contribute to this phenomenon. Transthyretin amyloidosis, which is resistant to degradation, can be deposited in locations within the CSF circulation, including the meninges, blood vessel walls, and subarachnoid granules (15). Additionally, nerve thickening observed in ultrasonography can also lead to obstruction and elevated protein content.

Anti-GT1a antibody positivity has been observed in different diseases, with binding sites mainly in the oculomotor, cranial, and vagus nerves (16). This contrasts with the peripheral nerve manifestations observed in this patient. The EMG presentation is also somewhat disorienting, as peripheral nerve damage in ATTRv is predominantly axonal. Furthermore, demyelination-oriented manifestations such as slowed conduction velocities are common in the EMG tests of patients with ATTRv. However, axonal damage is generally more frequent and severe than demyelination damage. A reduction in the amplitude of the ulnar nerve action potential (UAP) and peroneal nerve action potential (PAP) to below 5.4 mV and 3.95 μV, respectively, could indicate the presence of hereditary ATTRv-PN (17).

Notably, pathological biopsies may be misleading due to the scattered deposition of amyloid deposits, which can result in neuropathologic biopsies failing to yield positive results. Additionally, the pathological process of demyelination and myelin regeneration in this disease may erroneously lead to the diagnosis of demyelinating peripheral neuropathy. Amyloid deposits may not be completely identified as TTR deposits, and in a few cases, they can be labelled by anti-light chain antibodies, resulting in a false diagnosis (18). This indicates the necessity of genetic testing for accurate diagnosis. In patients with a diagnosis of CIDP and inadequate response to pulse therapy of methylprednisolone or immunoglobulin, it is important to consider the possibility ATTRv, even in the presence of protein cell detachment, positive antibodies to peripheral neuropathies, and decreased EMG nerve conduction velocities.

A pathological biopsy is crucial for diagnosing this disease. Although it is preferable to directly biopsy the affected nerve tissue, obtaining a sample is challenging and potentially harmful. Additionally, the random distribution of amyloid deposits in the nerve tissue may result in a low positive detection rate. Abdominal fat biopsy has been demonstrated to have a high detection rate for ATTRv with neurologic manifestations, exhibiting both good specificity and sensitivity (19). In this instance, we employed an abdominal adipose tissue biopsy, which is less invasive, more tolerable, and more readily accepted by the patient. Given the random distribution of amyloid deposits, we opted for samples from multiple locations in the abdomen to increase the positivity rate, and we were fortunate to obtain a positive result. Consequently, abdominal multisite biopsy represents a highly feasible, minimally invasive, and highly recommended option for patients requiring a pathological biopsy.

## 5 Conclusion

We present three cases in a Chinese four-generation family diagnosed with Gly103Arg variant-associated ATTRv and collect clinical data of 41 patients with the Gly103Arg variant from 18 unrelated families. The peripheral neurologic presentations may be easily misdiagnosed as CIDP. It is therefore crucial to inquire about family history and ocular diseases to minimize the risk of misdiagnosis. During ophthalmological consultations, patients with vitreous opacity should be queried about their family history. Thus, for Chinese especially in southern area with a family history of vitreous opacities, server axonal damage and ineffective pulse therapy of methylprednisolone, this mutation-associated ATTRv should be considered. Timely pathological biopsy and genetic testing can assist in the early diagnosis of this condition. Finally, abdominal adipose tissue biopsy is a convenient, inexpensive, and minimally invasive means of confirming the diagnosis, with multiple sampling sites increasing the positive rate.

## Data Availability

The datasets presented in this study can be found in online repositories. The names of the repository/repositories and accession number(s) can be found in the article/supplementary material.
